# In the Blood

**Published:** 1859-03

**Authors:** 


### IN THE BLOOD.

Dyed in the wool, radical, inherent, of a piece, these are various forms of expression intended to convey one and the same idea, to wit—a part of a chip of the same block. But by the expression "in the blood," we desire here to convey a moral idea, by the aid of a medical phrase; an idea repudiated by multitudes, abhorred by not a few, but true for all that, as the following narration may illustrate: A city merchant wanted a small boy in his store; one aged ten years was highly recommended by a lady, who guaranteed his good conduct, she having befriended and aided the family materially, for several years since their arrival in this country. The youth was not known to have been in a place of trust before. He proved to be diligent and attentive; small pieces of money were brought to the proprietor from time to time, as picked up from the floor in sweeping out, and there was an evident effort to please. Within a week of his entrance stolen property and money were found in his pocket, which at the instant before discovery, he declared contained nothing whatever, but it did contain the proprietor's pocket book, with money, papers, &c. Here was a systematic effort of a mere child, began from the very first day of entering the store, by an appearance of strict honesty and integrity in trifling matters, to throw the proprietor off his guard, to enable the child to steal from the shelves and cash box without suspicion. We personally know the facts of the case, and can account for such precociousness in crime, such adeptness in deception, such facility and aptitude for perpetrating thefts, in no other way, than that both father and mother were thieves and liars, and had never been any thing else, having been indoctrinated thus for perhaps long generations preceding. We know that persons are born with the physical characteristics of their parents—born with their parents' diseases. Napoleon's mental nature was impregnated from his mother before his birth, when she rode by her warrior husband at the head of armed bands for days and weeks and months together; while at the same time, he inherited the disease of his father, and likewise perished with it. It is notorious that three-fourths of the idiotic are born of parents, one or both of whom are drunken; shadowing the state of mind of the parent, bestial, stupid, low, at the instant of conception, as the mould in which the child is cast. Some practical use may be made of these things, but not we presume, until the human mind becomes more generally, more thoroughly, more supremely religious from principle, high, uniform, abiding.—

What, therefore, physiology teaches of corporeal man, the Bible repeats as to his moral nature, in the stern declaration that "the wicked are estranged from the womb, they go astray as soon as they be born, speaking lies." That it is just as natural for man to sin, as it is for the sparks to fly upward, or for a duck to take to the water the instant it breaks from the shell. Sin and crime bring destitution, disease and death. From another direction then, we come to the practical conclusion, stated in the last number, that the time for impressing the future child with greatest certainty with a high moral character, is during the months preceding its birth, just as certainly as a high state of physical health kept up during gestation, is one of the most certain means of ensuring a good constitution to the coming being.

It, therefore, seems to follow, that all modes of human reform, in order to be successful, must be founded on truth, and that the million plans which have been spawned forth on the world, with only a butterfly life, have had their foundations laid in error, in false doctrine, and that false doctrine has colored almost every system of human amelioration which has ever been presented; it is the doctrine of human perfectibility as opposed to human depravity, innate and total: a depravity not equally deep as to all, but a depravity of varying shades, pervading all, from the new born infant to the centennarian. Owen of Lanark, Cabot of Paris, Communism and the Philanstery, all founder here; and their defeated glorifiers now crimson not to confess that their systems are only adapted to the unselfish; which means really, that to succeed, they must have perfect men to begin with: but ask them how they will make men perfect, and they are either as dumb as the ass, or utter incoherent ravings about education and the elevation of the masses. Then, philosophers, so called, may blunder and flounder and prate as they please, but it all comes to this at last, that the very first step towards human elevation is in human abasement; each man for himself must see and feel and acknowledge that he is a poor, weak, miserable sinner, and then, in the light of the bible, look for help in the direction of Him, who is able to elevate and save all who, while looking, believe and live.

